# Photoreceptor-targeted engineered exosomes encapsulating black phosphorus quantum dots for alleviating photoreceptor degeneration

**DOI:** 10.1186/s12951-026-04763-x

**Published:** 2026-07-08

**Authors:** Jia Liang, Xiangqing Hei, Lu Chen, Zhenhua Zou, Yuke Ji, Lujia Feng, Wangting Li, Yijing Zhuang, Bingyu Bai, Renxue Liang, Yunyun Zou, Pan Xiao, Qiang Lin, Huiyan Zheng, Dong Fang, Wei Chi, Shaochong Zhang

**Affiliations:** 1 Shenzhen Eye Hospital, Shenzhen Eye Medical Center, Southern Medical University, 18 Zetian Road, Shenzhen, 518040 Guangdong China; 2 Shenzhen Eye Hospital, Jinan University, 18 Zetian Road, Shenzhen, 518040 Guangdong China

**Keywords:** Photoreceptor degeneration, Black phosphorus quantum dots, Exosome, Targeted delivery, Mitochondrial apoptosis

## Abstract

Photoreceptor degeneration is a major cause of irreversible visual impairment worldwide, most notably in retinitis pigmentosa (RP). Here, we developed a dual-targeted therapeutic nanoplatform by loading black phosphorus quantum dots (BPQDs) into human dental pulp mesenchymal stem cell-derived exosomes (hDPSC-Exos) and surface-modifying with cell-penetrating peptide transactivated transcription (TAT) and photoreceptor-specific peptide MH42, namely TAT/MH42-engineered black phosphorus quantum dot-loaded exosomes (M/T-BPQD@Exos). M/T-BPQD@Exos showed typical exosomal morphology and good uniformity. In vitro, M/T-BPQD@Exos effectively protected 661 W photoreceptor cells against N-methyl-N-nitrosourea (MNU) injury. In vivo, M/T-BPQD@Exos specifically accumulated in the outer nuclear layer (ONL) and significantly preserved retinal structure and visual function in MNU-induced photoreceptor degeneration rats. Biosafety assessment confirmed no obvious systemic toxicity and inflammation. Mechanistically, M/T-BPQD@Exos alleviated calcium overload, maintained mitochondrial integrity, and suppressed mitochondrial apoptosis by regulating Bcl-2, Bax, and cleaved caspase-3. This study provides a safe and precise strategy for photoreceptor protection in retinal degenerative diseases.

**Graphical Abstract:**

**Figure:**
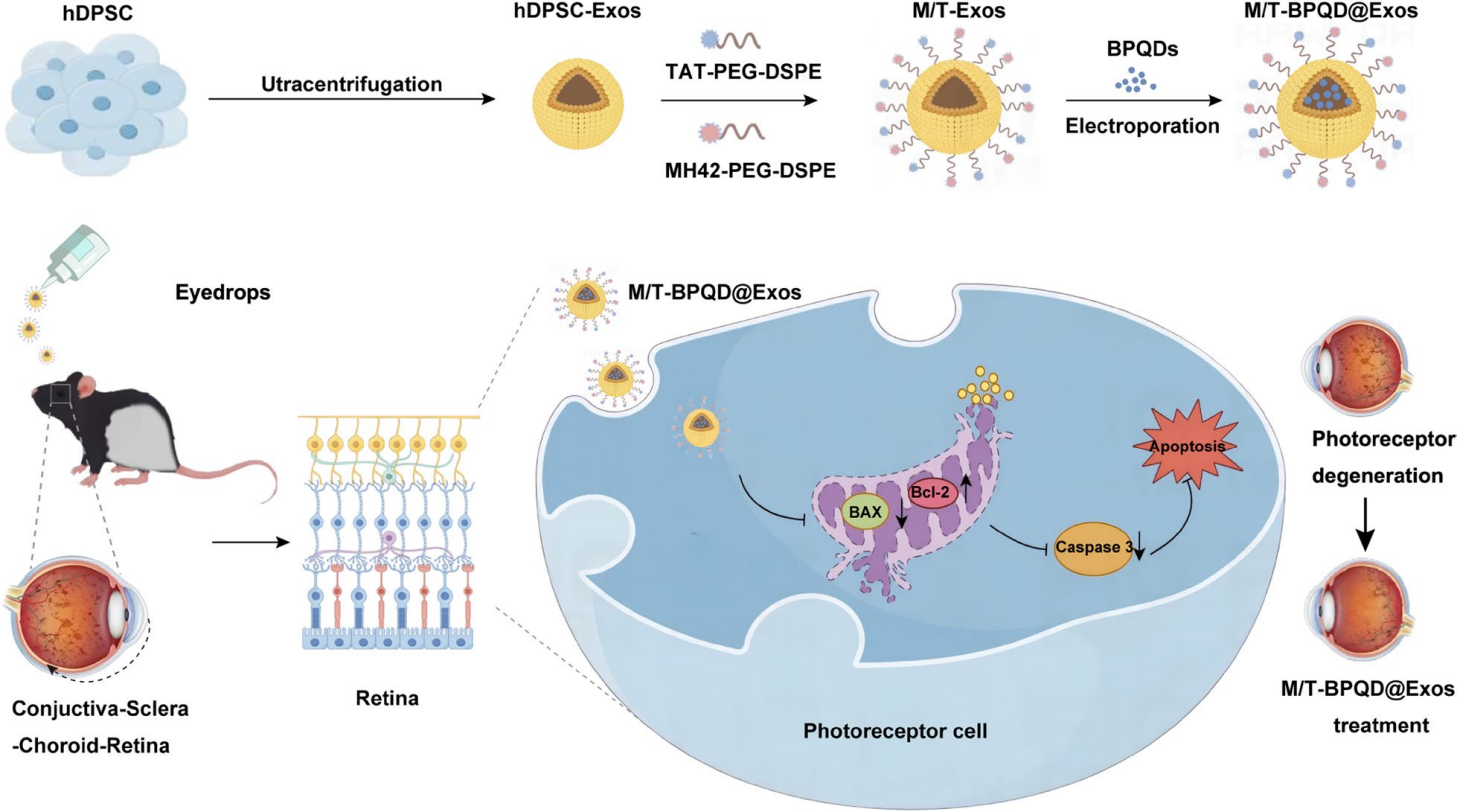

**Supplementary Information:**

The online version contains supplementary material available at 10.1186/s12951-026-04763-x.

## Introduction

 Photoreceptor degeneration, most notably in retinitis pigmentosa (RP), is a leading cause of irreversible blindness worldwide, affecting over 1 in 3500 individuals globally [1]. Among preclinical models mimicking photoreceptor loss, N-methyl-N-nitrosourea (MNU)-induced retinal degeneration is widely used for its high reproducibility and pathological alignment with human RP. MNU selectively damages rod and cone photoreceptors by apoptosis, ultimately resulting in retinal outer nuclear layer (ONL) thinning and irreversible visual function impairment [2, 3]. Despite decades of therapeutic exploration, current strategies remain inadequate: oral antioxidants only slow disease progression without reversing established damage, while viral vector-based gene therapy is limited by immunogenicity, packaging capacity constraints, and potential insertional mutagenesis [1]. Thus, there is an urgent need for a non-viral, tissue-compatible targeted delivery system that can simultaneously protect photoreceptors and modulate core pathological pathways.

Extracellular vesicles (EVs) have emerged as ideal natural carriers for ocular drug delivery, thanks to their excellent biocompatibility, low immunogenicity, and inherent ability to cross the blood-retinal barrier (BRB) [4]. Among various EV sources, human dental pulp mesenchymal stem cell-derived exosomes (hDPSC-Exos) stand out with superior biological properties—they are easily accessible, non-invasively isolated, and exhibit more potent anti-inflammatory, anti-apoptotic, and neuroprotective effects than many other mesenchymal stem cell (MSC) sources, making them particularly promising for retinal repair and photoreceptor protection [5, 6]. Notably, hDPSC-Exos have been widely documented to promote neuroprotection in retinal degenerative diseases, glaucoma, and cerebral ischemia/reperfusion injury [7, 8]. Meanwhile, exosomes derived from retinal pigment epithelium (RPE) cells, Müller glia, and retinal progenitor cells have also been explored for targeted intraocular delivery and retinal protection [9, 10].

However, unmodified exosomes suffer from two critical bottlenecks that severely limit their therapeutic efficacy: insufficient penetration across the dense retinal layers and lack of specific targeting to photoreceptors, resulting in off-target distribution, rapid clearance, and low local concentration in the ONL [11, 12]. To overcome these limitations, peptide-mediated surface engineering has become a powerful strategy to precisely program exosomal delivery. Among functional peptides, the TAT (Transactivated transcription) peptide is a gold-standard cell-penetrating peptide with exceptional membrane-translocation ability [13]. TAT enables cargos to efficiently cross cell membranes, corneal epithelium, conjunctival–scleral barriers, and the BRB, dramatically improving intraretinal diffusion, deep layer penetration, and long-term retention [14, 15]. Complementarily, MH42 peptide is a well-validated photoreceptor-specific targeting ligand that selectively binds to surface receptors on rod and cone photoreceptors, enabling precise enrichment of therapeutics in the ONL—the primary site of photoreceptor degeneration [16, 17]. Dual modification with TAT and MH42 creates a synergistic delivery system that first penetrates all retinal layers and then specifically anchors to photoreceptors, solving the dual challenges of poor penetration and off-target distribution simultaneously.

Black phosphorus quantum dots (BPQDs), a novel two-dimensional nanomaterial, have recently shown great potential in ophthalmology due to their unique physicochemical properties: their ultra-small size (~ 5–10 nm) enables efficient penetration through retinal layers, and their surface hydroxyl groups endow them with dual functions of iron chelation and reactive oxygen species (ROS) scavenging—two key mechanisms to inhibit MNU-induced photoreceptor ferroptosis [18, 19]. Furthermore, BPQDs are biodegradable, decomposing into non-toxic phosphate ions that are metabolized by the body, avoiding long-term retinal accumulation and potential cytotoxicity [18]. Nevertheless, free BPQDs administered systemically or locally face rapid clearance in the ocular microenvironment and non-specific distribution to non-target cells (e.g., RPE or choroidal cells), significantly reducing their therapeutic efficacy for photoreceptor degeneration [20]. Encapsulation within engineered exosomes can protect BPQDs from degradation, prolong their retention, and guide their precise delivery to photoreceptors.

Based on the above rationales, we rationally designed and constructed a dual-targeted engineered exosomal nanoplatform (M/T-BPQD@Exos) by co-modifying hDPSC-derived exosomes with TAT and MH42 peptides and encapsulating BPQDs as the therapeutic core. We hypothesized that this integrated system would: (1) penetrate deep into the retina via TAT-mediated membrane translocation; (2) specifically target photoreceptors via MH42-mediated recognition; (3) release BPQDs locally to suppress oxidative stress, calcium dyshomeostasis, and mitochondrial apoptosis; (4) preserve photoreceptor structure and function, and improve visual in MNU-induced degeneration. This study aimed to characterize the physicochemical properties, evaluate in vitro targeting and cytoprotective effects, validate in vivo therapeutic efficacy and biosafety, and elucidate the underlying molecular mechanisms of M/T-BPQD@Exos. By combining barrier-penetrating delivery, precise photoreceptor targeting, and multifunctional neuroprotection, this platform represents a promising and translatable strategy for treating photoreceptor degenerative diseases. 

## Materials and methods

### Materials and reagents

Normal Retinal dystrophy (RDY) rats (8–10 weeks old, male, 250–300 g) were purchased from Xian Provincial Experimental Animal Research Center (Xian, China). N-methyl-N-nitrosourea (MNU) was purchased from Aladdin Biochemical Technology Co., Ltd. (Shanghai, China). BPQDs flakes were obtained from Delta BioMaterials Co., Ltd. (Guangzhou, China) and 1,2-distearoyl-sn-glycero-3-phosphoethanolamine-N-[biotinyl (polyethylene glycol)-2000] (DSPE-PEG2000-Biotin) was obtained from Xi’an Ruixi Biological Technology Co., Ltd. (Xi’an, China). Human dental pulp mesenchymal stem cells (HUXDP-01001) were purchased from OriCell (Cyagen Biosciences Inc., Guangzhou, China). Photoreceptor-targeting MH42-PEG-DSPE fusion peptide (sequence: SPALHFLGGGSC) and the TAT-PEG-DSPE peptide (GRKKPRQRRRPPQ) were synthesized and purified by ChinaPeptides (Shanghai, China) and molecular weight confirmed by matrix-assisted laser desorption/ionization time-of-flight mass spectrometry (MALDI-TOF MS). 4’,6-diamidino-2-phenylindole (DAPI), propidium iodide (PI) and Fluo-4 acetoxymethyl ester (Fluo4 AM) calcium indicator were obtained from Beyotime Biotechnology (Shanghai, China). Radioimmunoprecipitation assay (RIPA) lysis buffer, bicinchoninic acid (BCA) protein quantification kit, enhanced chemiluminescence (ECL) kit, and terminal deoxynucleotidyl transferase dUTP nick end labeling (TUNEL) apoptosis detection kit were purchased from Beyotime Biotechnology (Shanghai, China). Annexin V-FITC/PI apoptosis detection kit was obtained from BD Biosciences (Franklin Lakes, NJ, USA). Primary antibodies included: anti-CD63 (ab217347), anti-CD81 (ab219219), anti-Calnexin (ab22595), anti-Bax (ab182733), anti-caspase-3 (ab2302), anti-γ-H2AX (ab26350), purchased from Abcam (Cambridge, UK); anti-Bcl-2 (12789-1-AP) purchased from ProteinTech (Wuhan, China). Horseradish peroxidase (HRP)-conjugated secondary antibodies were obtained from Beyotime Biotechnology (Shanghai, China).

### Isolation and Purification of hDPSC-Derived Exosomes

Human dental pulp mesenchymal stem cell (hDPSC) culture supernatants were collected for exosome isolation using differential ultracentrifugation. Briefly, supernatants were centrifuged at 300 × g for 10 min, 2000 × g for 20 min, and 10,000 × g for 30 min at 4 °C to remove cell debris and microvesicles. The supernatant was then filtered through a 0.22 μm filter and ultracentrifuged at 100,000 × g for 70 min using a Beckman SW 41 Ti rotor (Beckman Coulter, Brea, CA, USA). The pellet was resuspended in sterile PBS and stored at − 80 °C for long-term preservation. Exosome morphology was observed under a transmission electron microscope (TEM, JEM-2100, JEOL, Tokyo, Japan). Size distribution and concentration were measured using nanoparticle tracking analysis (NTA, ZetaView PMX 110, Particle Metrix, Meerbusch, Germany). Exosome marker proteins (CD63, CD81) and negative marker Calnexin were identified by Western blot.

### Preparation of Dual-Targeted M/T-BPQD@Exos

BPQDs were first fabricated via liquid exfoliation, where 50 mg of BP flakes were dispersed in 10 mL of N-methylpyrrolidone (NMP) and sonicated in an ice bath at 300 W for 4 h with a 5 s on / 5 s off pulse cycle using a probe sonicator. The mixture was then centrifuged at 5000 × g for 15 min to remove unexfoliated residues, and the supernatant containing BPQDs was dialyzed against PBS (MWCO 3.5 kDa) for 24 h to eliminate organic solvent. The quantum yield of BPQDs in PBS was measured to be approximately 12%, which is consistent with values reported in previous literature [21]. For the construction of dual-targeted engineered exosomes, hDPSC-derived exosomes were incubated with TAT-PEG-DSPE and MH42-PEG-DSPE at a mass ratio of 1:1 (10 µg each per 100 µg exosomal protein) at 37 °C for 30 min with gentle shaking to insert the dual functional peptides into the exosomal membrane via hydrophobic insertion [22]. Unbound peptides were removed by another round of ultracentrifugation at 100,000 × g for 70 min. Subsequently, the peptide-modified exosomes at a concentration of 1 × 10¹¹ particles/mL were mixed with the prepared BPQDs (final concentration 200 µg/mL) in PBS, and electroporation was performed at 150 V and 900 µF with a single pulse using a Gene Pulser Xcell system to load BPQDs into the exosomal lumen. After incubation on ice for 30 min to allow membrane recovery, free BPQDs were removed by ultracentrifugation at 100,000 × g for 70 min.The final dual-targeted M/T-BPQD@Exos were resuspended in sterile PBS and stored at − 80 °C until further use to avoid repeated freeze-thaw cycles. A fresh aliquot was thawed immediately before each administration and used directly.

## Characterization of M/T-BPQD@Exos

### Morphology, particle size and zeta potential characterization

The morphology of M/T-BPQD@Exos was observed by transmission electron microscopy (TEM) after fixation with 2.5% glutaraldehyde at 4 °C for 2 h, deposition onto 200-mesh copper grids, and negative staining with 2% phosphotungstic acid (pH 7.0) for 5 min; excess dye was removed with filter paper, grids were air-dried at room temperature, and TEM images were acquired at 120 kV, with particle size measured using ImageJ software (*n* = 100 particles per sample). Size distribution, and polydispersity index (PDI) were determined by nanoparticle tracking analysis (NTA) at 25 °C, samples were diluted to 1 × 10⁸ particles/mL in PBS, measured in triplicate (60 s per test), and average values were recorded. Zeta potential of the M/T-BPQD@Exos was quantified with Zetasizer (Malvern Panalytical, UK) at 25.0 °C using PBS as dispersion medium.

### Western blot analysis of exosome markers

For Western blot analysis, total protein was extracted using RIPA lysis buffer (Beyotime) containing protease inhibitors, quantified with a BCA assay kit (Thermo Fisher Scientific), and 20 µg protein per lane was separated by 12% sodium dodecyl sulfate-polyacrylamide gel electrophoresis (SDS-PAGE) and transferred to PVDF membranes; membranes were blocked with 5% non-fat milk for 1 h at room temperature, incubated overnight at 4 °C with primary antibodies against CD63 (1:1000), CD81 (1:1000), and Calnexin (1:1000; negative control), washed with Tris-buffered saline with Tween (TBST), incubated with HRP-conjugated secondary antibody (1:5000, Beyotime) for 1 h at room temperature, and visualized using an ECL chemiluminescence kit (Millipore), with band gray values analyzed using ImageJ software.

### In vitro release assay

In vitro release profile of BPQDs from M/T-BPQD@Exos was determined via ultracentrifugation separation method [23]. Briefly, 5 mL M/T-BPQD@Exos suspension (containing 1 mg loaded BPQDs) was mixed with 10 mL pH 7.4 PBS and incubated at 37 °C with constant shaking. At predetermined time points (12, 24, 36, 48, 60, 72 h), 500 µL supernatant was collected after ultracentrifugation (20,000 g, 20 min, 4 °C), and an equal volume of pre-warmed fresh PBS was supplemented to maintain constant system volume. The phosphorus content in supernatant was quantified by ICP-MS to calculate cumulative BPQD release percentage.

### Stability of M/T-BPQD@Exos

The standard storage temperature for M/T-BPQD@Exos was set at − 80 °C. Short-term stability was evaluated by storing samples at 4 °C for 7 days. For long-term stability, samples were preserved at − 80 °C for 3 months. An accelerated stability test was performed at 37 °C for 24 h. At predetermined time points, particle size and PDI were measured to monitor the dispersion state of the samples.

## Cell culture and in vitro targeting and cytoprotection assays

The mouse photoreceptor cell line 661 W was cultured in RPMI 1640 medium supplemented with 10% exosome-depleted fetal bovine serum (FBS) at 37 °C in a humidified atmosphere containing 5% CO₂. For the cellular uptake assay, 661 W photoreceptor cells and non-target 293T cells were incubated with M/T-BPQD@Exos, and intracellular fluorescence signals were observed and recorded using a confocal laser scanning microscope (CLSM, LSM 880, Zeiss, Oberkochen, Germany). For cytoprotection evaluation, 661 W cells were treated with 400ug/ml MNU for 5 h in the presence or absence of M/T-BPQD@Exos, and cell viability was quantitatively assessed by DAPI/PI staining, cell counting, and survival rate analysis.

## In vivo therapeutic efficacy in MNU-induced photoreceptor degeneration rat model

### Establishment and treatment of MNU-induced retinal degeneration model

RDY rats were randomly divided into 4 groups (*n* = 6 per group): (1) Normal group (no MNU injection); (2) M/T-BPQD@Exos single treatment group; (3) MNU group (MNU injection + PBS treatment); (4) M/T-BPQD@Exos group (MNU injection + M/T-BPQD@Exos treatment). The MNU model was established by a single intraperitoneal injection of MNU (40 mg/kg) dissolved in 0.9% NaCl (pH adjusted to 7.0 with NaOH). All eye drop administrations were performed under anesthesia using a stereomicroscope to ensure precise delivery. After administration, the eyelid was gently closed for 10 s to avoid solution loss and improve ocular retention. Rats were administered topical eye drops containing 2 µL of PBS or M/T-BPQD@Exos twice a day (b.i.d.). The twice-daily dosing regimen was selected given the sustained retinal retention of the nanoparticles beyond 24 h, clinical routine practice, good efficacy, high biosafety and previous reports of topical ocular delivery systems for retinal diseases [24, 25].

### In vivo retinal distribution and retention analysis

M/T-BPQD@Exos were labeled with Cy5 fluorescent dye for in vivo tracking. Although BPQDs have intrinsic fluorescence, their intensity is relatively weak and easily interfered by ocular autofluorescence; thus, exogenous fluorescent labeling was used to achieve a higher signal-to-noise ratio. At 3 h, 6 h, 24 h, 48 h, 72 h, and 168 h after topical eye drop administration, retinal fluorescence signals were observed using an in vivo imaging system (IVIS, PerkinElmer, Waltham, MA, USA).

### Evaluation of visual function by electroretinography (ERG) and morris water maze test

At 3 and 7 days after treatment, rats were dark-adapted for 12 h, then anesthetized with intraperitoneal injection of sodium pentobarbital (50 mg/kg). Pupils were dilated with 1% tropicamide eye drops, and ERG was performed using a Visual Electrophysiology System (RetiPort 300, Roland Consult, Brandenburg, Germany) with a rodent corneal electrode. Scotopic ERG (dark-adapted) was recorded with a stimulus intensity of 0.1 cd·s/m², and the amplitudes of a-wave (from baseline to negative peak) and b-wave (from negative peak to positive peak) were measured. Spatial cognitive function was evaluated using the Morris water maze test. The escape latency, swimming distance, platform crossing times, and time spent in the target quadrant were recorded and analyzed.

### Retinal morphological analysis by optical coherence tomography (OCT), Hematoxylin-Eosin (HE) staining and transmission electron microscopy (TEM)

At 7 days after treatment, in vivo retinal morphology was evaluated by OCT (Spectralis HRA + OCT, Heidelberg Engineering, Heidelberg, Germany) under anesthesia. Standard linear B-scans centered on the optic disc were acquired. The thickness of the ONL was measured at 0.5 mm, 1.0 mm, and 1.5 mm from the optic disc (*n* = 3 measurements per eye). After OCT examination, rats were euthanized, and eyeballs were enucleated and fixed in 4% paraformaldehyde for 24 h. Eyeballs were dehydrated with gradient ethanol, embedded in paraffin, and sectioned into 5 μm-thick slices (passing through the optic disc). Slices were stained with HE, and images were acquired using a light microscope (BX53, Olympus, Tokyo, Japan). Retinal morphology and histological features were assessed by hematoxylin-eosin (HE) staining. The thickness of the ONL was measured at distances of ± 800, ±1600, ± 2400, and ± 3200 μm from the optic nerve head (ONH). Additionally, the ONL thickness and the number of ONL cells within the retinal region spanning from − 800 to 800 μm around the ONH were evaluated using ImageJ 1.47 software. For photoreceptor ultrastructural observation, retinal tissues were fixed in 2.5% glutaraldehyde in 0.1 M phosphate buffer (pH 7.4) at 4 °C overnight, post-fixed with 1% osmium tetroxide for 1 h, and then dehydrated through a graded ethanol series. After embedding in epoxy resin, ultra-thin Sect.  (70 nm) were cut using an ultramicrotome, stained with uranyl acetate and lead citrate, and examined with a transmission electron microscope (HITACHI HT 7800, Hitachi, Japan) operated at 80 kV. Nuclear pyknosis and mitochondrial morphology (including swelling and cristae disruption) were evaluated to assess photoreceptor damage.

## Biosafety evaluation

### Systemic biosafety assessment

At 7 days post-treatment, blood samples were collected for routine blood tests and serum biochemical analysis. Hematological indexes included monocytes (Mon), lymphocytes (Lymph), granulocytes (Gran), and red blood cells (RBC) for evaluating the systemic inflammation response. Liver and renal function markers, including alanine transaminase (ALT), aspartate transaminase (AST), blood urea nitrogen (BUN), and creatinine (CREA) were examined to evaluate systemic organ toxicity. Furthermore, serum levels of inflammatory cytokines (TNF-α, IL-1β, IL-6, and IFN-γ) were quantified by enzyme-linked immunosorbent assay (ELISA) to assess immunogenicity of hDPSC-derived exosomes and inflammation. Heart, liver, spleen, lung, kidney, and brain tissues were harvested for H&E staining to evaluate systemic toxicity and inflammatory infiltration.

### General ocular safety detection

For local ocular safety evaluation, we conducted macroscopic ocular observation, corneal fluorescence staining, intraocular pressure (IOP) detection and body weight monitoring. Aqueous humor was collected for intraocular cytokine quantification by ELISA. Retinal tissues were split into two parts: one for H&E histopathological staining to observe local inflammatory infiltration, and the other homogenized to detect retinal tissue levels of TNF-α, IL-1β, IL-6 and IFN-γ using ELISA.

### Ocular irritation test

Ocular irritation was evaluated using a modified Draize scoring system (*n* = 3). Briefly, 2 uL of M/T-BPQD@Exos eye drops was instilled into the conjunctival sac of one eye, and the contralateral eye receiving isotonic saline was set as self-control. Under slit lamp and naked-eye observation, corneal opacity, iris lesion as well as conjunctival hyperemia, chemosis and discharge were scored at 1, 6, 24, 48, 72 h and 7d post-administration. The total irritation scores remained consistently ≤ 3 at all time points, and all mild transient ocular manifestations fully recovered within 24 h, demonstrating negligible ocular irritation and favorable biocompatibility for topical application.

## Transcriptome sequencing and bioinformatics analysis

Total RNA was extracted from retinal tissues using a standard kit. RNA-seq was performed on the DNBSEQ platform (BGI, Shenzhen, China). Differentially expressed genes (DEGs) were analyzed using DESeq2. Gene Ontology (GO) and Kyoto Encyclopedia of Genes and Genomes (KEGG) enrichment analyses were performed. Protein–protein interaction (PPI) networks and hub genes were identified using STRING and Cytoscape software.

## Western blot analysis

Total protein was extracted from retinal tissues and 661 W cells using RIPA lysis buffer (Beyotime, Shanghai, China) supplemented with protease inhibitors. Protein concentration was determined using a BCA protein assay kit (Thermo Fisher Scientific, Waltham, MA, USA). Equal amounts of protein (20 µg per lane) were separated by 12% SDS-PAGE and transferred onto polyvinylidene fluoride (PVDF) membranes (Millipore, Billerica, MA, USA). After blocking with 5% non-fat milk at room temperature for 1 h, the membranes were incubated overnight at 4 °C with primary antibodies: anti-CD63 (1:1000, Abcam, ab217347), anti-CD81 (1:1000, Abcam, ab219219), anti-Calnexin (1:1000, Abcam, ab22595), anti-Bcl-2 (1:1000, ProteinTech, 12789-1-AP), anti-Bax (1:1000, Abcam, ab182733), anti-cleaved caspase-3 (1:1000, Abcam, ab2302), and anti-γ-H2AX (1:1000, Abcam, ab26350). Subsequently, the membranes were incubated with HRP-conjugated secondary antibody (1:5000, Beyotime, Shanghai, China) for 1 h at room temperature. Protein bands were visualized using an ECL chemiluminescence kit (Millipore), and band gray values were quantified with ImageJ software.

## Statistical analysis

All data were presented as mean ± standard deviation (SD). Statistical analysis was performed using GraphPad Prism 9.0 software (GraphPad Software, San Diego, CA, USA). Differences between multiple groups were analyzed by one-way analysis of variance (ANOVA) followed by Tukey’s post-hoc test. *P* < 0.05 was considered statistically significant.

## Results

### Characterization, cytoprotection, and retinal targeting of dual-targeted M/T-BPQD@Exos

**Fig. 1:**
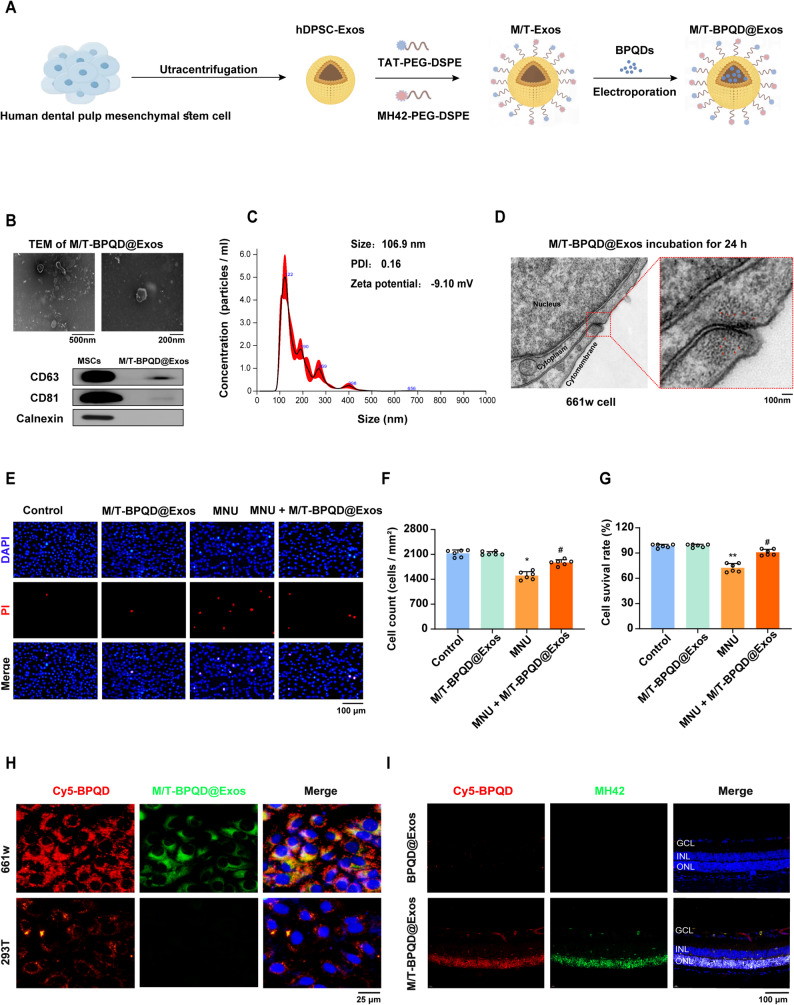
Characterization, cytoprotection, and retinal targeting of dual-targeted M/T-BPQD@Exos. (**A**) Schematic illustration of the fabrication of dual-targeted M/T-BPQD@Exos. (**B**) TEM images of M/T-BPQD@Exos and Western blot analysis of exosomal markers (CD63, CD81) and calnexin. (**C**) Size distribution and PDI of M/T-BPQD@Exos measured by NTA and Zeta potential was determined with Zetasizer. Data were repeated by three times, and SD was represented in red area. (**D**) TEM images showing the internalization of M/T-BPQD@Exos in 661 W cells after 24 h incubation. (**E**) Representative images of DAPI/PI staining in 661 W cells with different treatments. (**F-G**) Quantitative analysis of cell count and cell survival rate. (**H**) In vitro cellular uptake of M/T-BPQD@Exos in 661 W photoreceptor cells and non-target 293T cells, detected by immunofluorescence staining for Cy5-BPQD (red) and MH42 peptide on M/T‑BPQD@Exos (green). (**I**) In vivo retinal distribution of BPQD@Exos and M/T-BPQD@Exos at 24 h after topical ocular eye-drop administration in rats, with immunofluorescence staining for Cy5-BPQD (red) and MH42 targeting peptide conjugated on M/T-BPQD@Exos (green). M/T-BPQD@Exos, MH42/transactivated transcription peptide-modified black phosphorus quantum dot-loaded exosomes; TEM, transmission electron microscopy; PDI, polydispersity index; NTA, nanoparticle tracking analysis; DAPI,4’,6-diamidino-2-phenylindole; PI, propidium iodide. Scale bars: 500 nm, 200 nm (**B**); 100 nm (**D**); 100 μm (**E**); 25 μm (**H**); 100 μm (**I**). *p < 0.05, **p < 0.01 vs. Control; ^#^*p* < 0.05 vs. MNU group

Based on the CCK-8 results, we selected 1 × 10⁷ particles/mL as the optimal working concentration for M/T-BPQD@Exos. This dosage exerted the strongest cytoprotective effect against MNU-induced injury, and was superior to single BPQDs or hDPSC-Exos treatment (Supplementary Figure S1). We then comprehensively characterized the dual-targeted nanoplatform. As shown in Fig. 1A, M/T-BPQD@Exos were fabricated by loading BPQDs into hDPSC-Exos via electroporation, aftersurface modification with TAT-PEG-DSPE and MH42-PEG-DSPE. Structural characterization by TEM confirmed the typical cup-shaped morphology of M/T-BPQD@Exos. Western blot analysis verified the expression of exosomal markers CD63 and CD81 and the absence of calnexin, indicating high purity (Fig. 1B). NTA and zeta potential tests showed gradual changes in particle size, PDI and zeta potential during preparation, which confirmed successful BPQD loading and peptide modification (Supplementary Figure S2). The final M/T-BPQD@Exos displayed uniform size distribution with an average diameter of 106.90 nm via NTA, accompanied by a PDI of 0.16 and zeta potential of − 9.10 mV (Fig. 1C). TEM imaging following 24 h incubation further demonstrated efficient internalization of M/T-BPQD@Exos within 661 W cells (Fig. 1D). Stability tests showed that the formulation remained stable for 3 months at − 80 °C, 7 days at 4 °C and 24 h at 37 °C without obvious degradation or aggregation (Supplementary Table 1). The in vitro release test showed mild and continuous slow release of BPQDs: the cumulative release was 1.8% at 12 h, 4.2% at 24 h, 7.1% at 36 h and finally reached 8.6% at 72 h (Supplementary Figure S3). Cell experiments demonstrated that MNU significantly reduced 661 W cell viability, while M/T-BPQD@Exos obviously improved cell survival (Figs. 1E-G). In vitro cellular uptake assays confirmed strong specific binding of M/T-BPQD@Exos to 661 W photoreceptor cells, with negligible uptake in 293T cells (Fig. 1H). In vivo retinal distribution results proved that unmodified exosomes could not penetrate ocular barriers, while M/T-BPQD@Exos efficiently reached the retinal ONL. These results confirmed that TAT mediates barrier penetration (both corneal-scleral and retinal layers), and MH42 mediates photoreceptor-specific targeting, supporting the clinical translatability of topical eye drop administration (Fig. 1I).

### M/T-BPQD@Exos treatment ameliorates MNU-induced photoreceptor degeneration in vivo

**Fig. 2:**
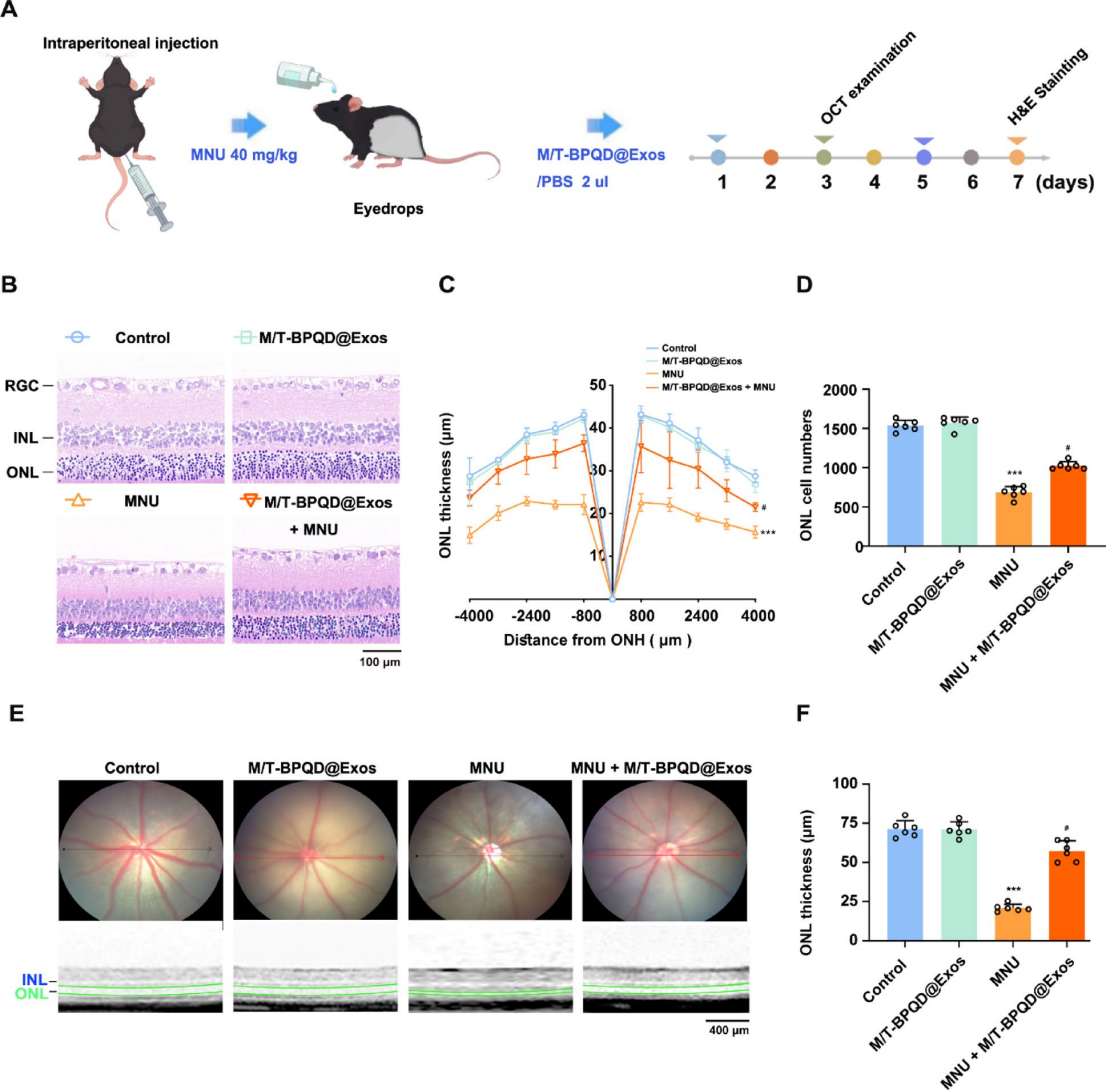
M/T-BPQD@Exos treatment ameliorates MNU-induced photoreceptor degeneration in vivo. (**A**) Schematic diagram of the verification of the therapeutic effect of M/T-BPQD@Exos in photoreceptor degeneration rat. (**B**) Representative photomicrographs of retinal slices stained with H&E. (**C-D**) The quantification of the thickness and cell numbers of ONL in MNU-injured and control rats, with or without M/T-BPQD@Exos administration. (**E**) Representative fundus images (upper row) and corresponding OCT images (lower row) of retinas in different groups. (**F**) The quantification analysis of ONL thickness based on OCT images in different groups. MNU: N-methyl-N-nitrosourea; H&E, Hematoxylin-eosin; RGC: retinal ganglion cell layer; INL: inner nuclear layer; ONL: outer nuclear layer; OCT, optical coherence tomography. Data are shown as the mean ± SD; ***p < 0.001vs. Control; ^#^*p* < 0.05 vs. MNU group. Scale bars: 100 μm (**B**), 400 μm (**E**)

A rat model was established via intraperitoneal injection of 40 mg/kg MNU, followed by topical eye drops of 2 µL PBS or M/T-BPQD@Exos twice daily, with retinal structure and function monitored over 7 days (Fig. 2A). H&E staining revealed that MNU caused severe thinning of the ONL and massive photoreceptor loss, while M/T-BPQD@Exos treatment significantly preserved ONL structure (Fig. 2B). Quantitative analysis showed that MNU caused a significant reduction in ONL thickness at all distances from the optic nerve head and cell number, which was significantly preserved by M/T-BPQD@Exos (Fig. 2C-D). Fundus photography and OCT examination further confirmed that MNU induced obvious retinal atrophy and ONL thinning, which was effectively alleviated by M/T-BPQD@Exos treatment **(**Fig. 2E-F**)**. Collectively, these results demonstrate that M/T-BPQD@Exos exerts protective effects against MNU-induced photoreceptor degeneration, preserving retinal structure and photoreceptor cell viability in vivo.

### M/T-BPQD@Exos preserves retinal visual function in MNU-induced photoreceptor degeneration rats

**Fig. 3:**
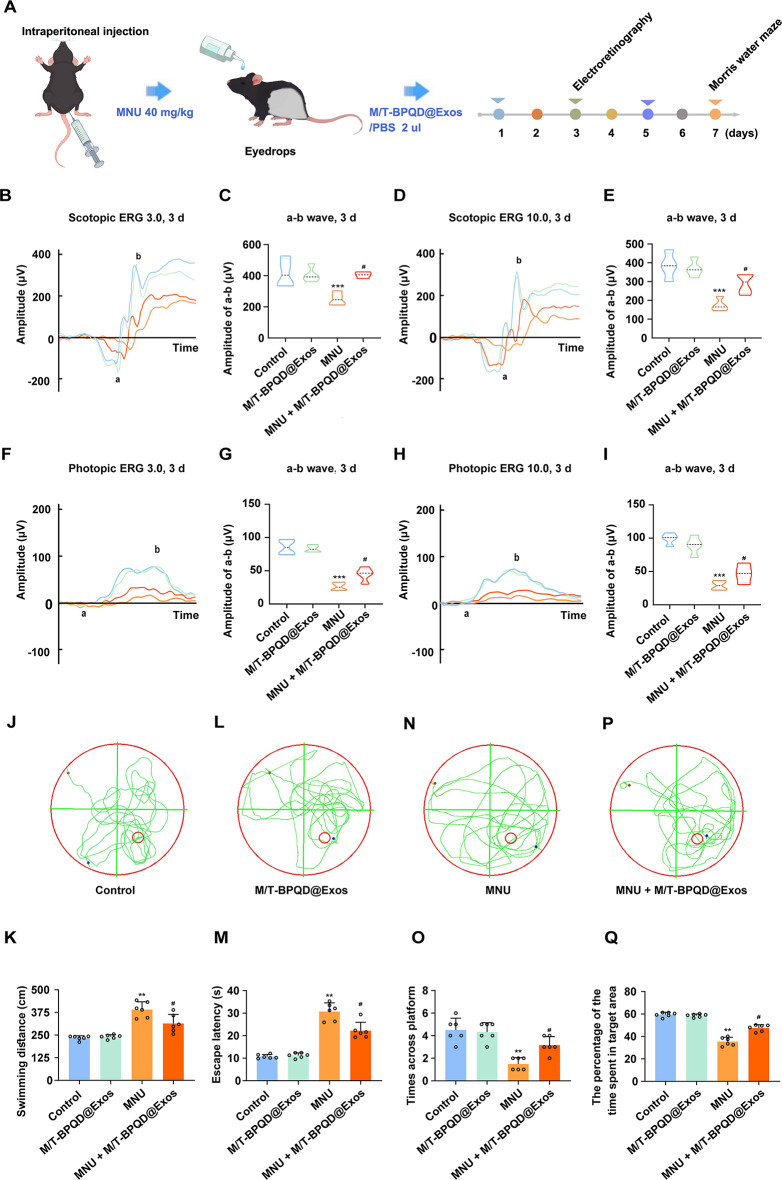
M/T-BPQD@Exos preserves retinal visual function in MNU-induced photoreceptor degeneration rats (**A**) Schematic diagram of the verification of the therapeutic effect of M/T-BPQD@Exos on visual function in RP rats. (**B-I**) Representative ERG waves detected in dark/light adaptation modes and the amplitude of the a-b waves. (**J-P**) The representative swim paths of the Morris water maze test in MNU-injured and control rats, with or without M/T-BPQD@Exos administration at 7 days post-treatment. (**K-Q**) The quantification analysis of swimming distance, escape latency, time spent in the target quadrant and percentage of time spent in the target area. MNU: N-methyl-N-nitrosourea; RP, retinitis pigmentosa; ERG, electroretinogram; Data are shown as the mean ± SD; **p < 0.01, ***p < 0.001vs. Control; ^#^*p* < 0.05 vs. MNU group.

After the photoreceptor degeneration rat model was established, rats receivedtopical eye drops of 2 µL M/T-BPQD@Exos or PBS, with retinal function evaluated at day 3 and day 7 post-treatment **(**Fig. 3A**)**. ERG analysis revealed that MNU treatment caused a dramatic reduction in a- and b-wave amplitudes at 3.0 and 10.0 cd·s/m²under both scotopic **(**Figs. 3B–E**)** and photopic **(**Fig. 3F–I**)** conditions, while M/T-BPQD@Exos significantly restored visual function. Morris water maze tests further supported the visual protective effects of M/T-BPQD@Exos **(**Fig. 3J–Q**)**. MNU-treated rats exhibited significantly increased swimming distance **(**Fig. 3K**)**, prolonged escape latency **(**Fig. 3M**)**, fewer platform crossings **(**Fig. 3O**)**, and less time spent in the target quadrant **(**Fig. 3Q**)**, all of which were notably improved by M/T-BPQD@Exos treatment. These behavioral results are consistent with ERG findings, confirming that M/T-BPQD@Exos effectively preserves visual function in vivo.

### Metabolic characteristics of M/T-BPQD@Exos in the eye and safety evaluation of M/T-BPQD@Exos in body

**Fig. 4:**
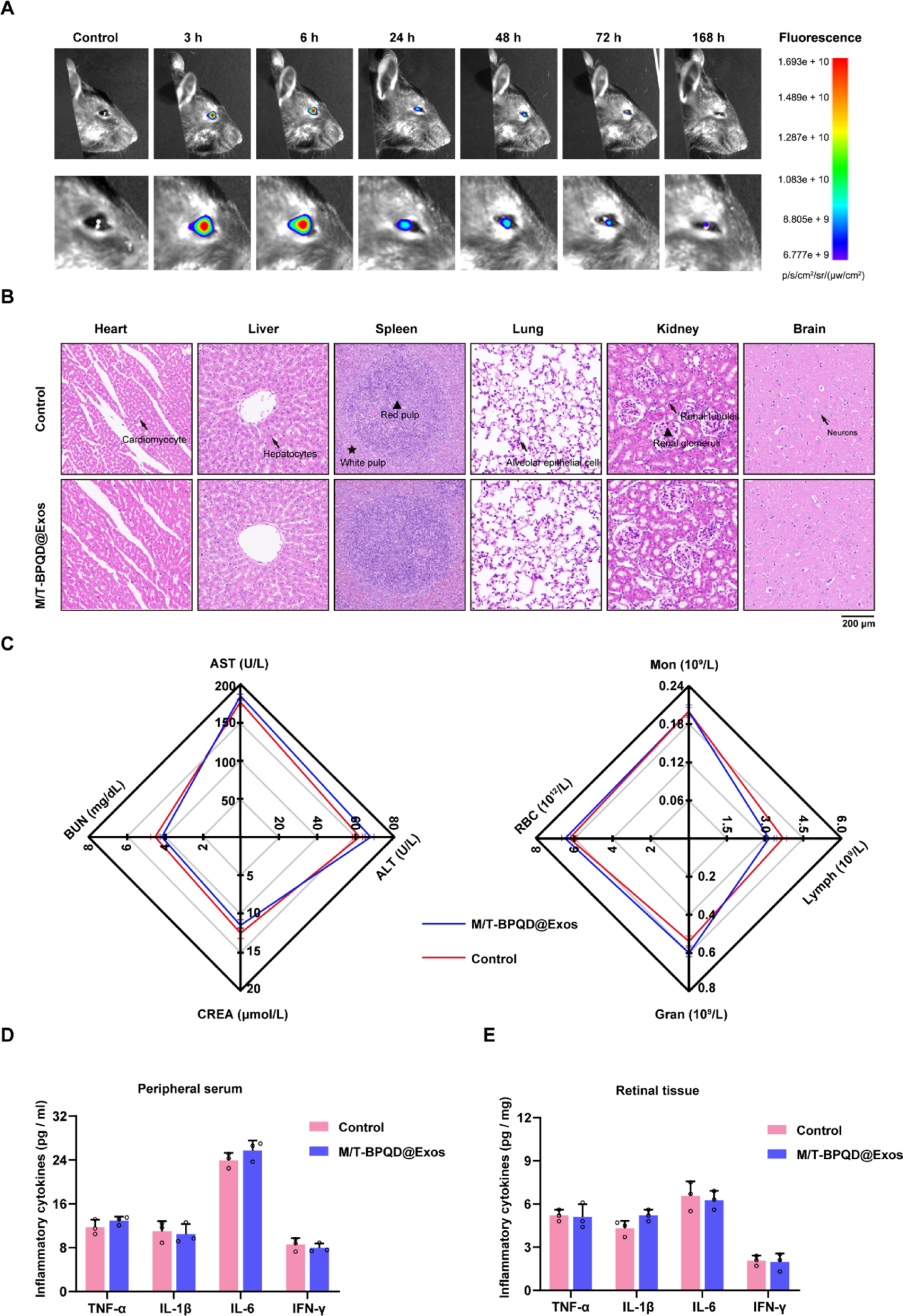
Metabolic characteristics of M/T-BPQD@Exos in the eye and safety evaluation of M/T-BPQD@Exos. (**A**) Fluorescence intensity of the Cy5-labeled M/T-BPQD@Exos in the eye detected by IVIS after topical ocular eye-drop administration. (**B**) H&E staining showing vital organs and key histological cellular/layer structures of the control and M/T-BPQD@Exos-treated rats. (**C**) Comparison of hepatic and renal function and routine blood parameters between the control and M/T-BPQD@Exos-treated rats. (**D-E**) Comparison of inflammatory cytokine levels in peripheral serum and retinal tissue between the control and M/T-BPQD@Exos-treated rats. IVIS, in vivo imaging system; ALT, alanine transaminase; AST, aspartate transaminase; BUN, blood urea nitrogen; CREA, creatinine; Gran, granulocytes; H&E, Hematoxylin and Eosin; Lymph, Lymphocytes; MON, monocytes; RBC, red blood cells. Scale bars: 200 μm  (**B**)

To evaluate the in vivo retinal retention, biodistribution, and biosafety of M/T-BPQD@Exos, in vivo fluorescence imaging, histological analysis, and serum biochemical/hematological assays were performed. In vivo fluorescence imaging showed that M/T-BPQD@Exos reached the retina at 3 h, peaked at 6 h, and could be detected for up to 168 h (7 days), showing excellent ocular retention (Fig. 4A). Histopathological analysis of major organs showed no systemic tissue injury or inflammation between M/T-BPQD@Exos treated and control rats. In both groups, the myocardium, liver, spleen, lung, kidney and brain presented normal histomorphology: orderly cardiomyocytes without necrosis or inflammation, intact hepatocytes with regular lobular structure, well-maintained splenic tissues, clear alveoli without congestion, normal renal glomeruli and tubules, and neurons free of swelling or karyopyknosis (Fig. 4B). Furthermore, blood routine, liver and renal function indexes all stayed within normal ranges (Fig. 4C). Serum, aqueous humor and retinal inflammatory cytokines (TNF-α, IL-1β, IL-6 and IFN-γ) in the M/T-BPQD@Exos group showed no significant differences compared to control group, indicating that M/T-BPQD@Exos did not trigger obvious systemic immune and inflammatory responses and confirming the low immunogenicity of hDPSC-derived exosomes (Fig. 4D and Supplementary Figure S4). To fully verify the absence of inflammation, we performed histological analysis of ocular and systemic tissues, corneal fluorescence staining and modified Draize scoring. No inflammatory infiltration, abnormal inflammatory markers or ocular lesions were observed in the treatment group. Using isotonic saline as control, we further evaluated ocular tolerability: the total modified Draize score remained ≤ 3, indicating no ocular irritation. No intergroup differences in IOP were detected. Animal body weight stayed stable, and no visible corneal or conjunctival inflammation occurred throughout treatment (Supplementary Figure S5). These results demonstrate that M/T-BPQD@Exos-based nanocarriers possess excellent retinal targeting, long-term retention, and favorable in vivo biosafety, supporting their potential for safe clinical translation in ocular disease treatment .

### Transcriptomic analysis of differential gene expression and functional enrichment in MNU-induced rats following M/T-BPQD@Exos treatment

**Fig. 5:**
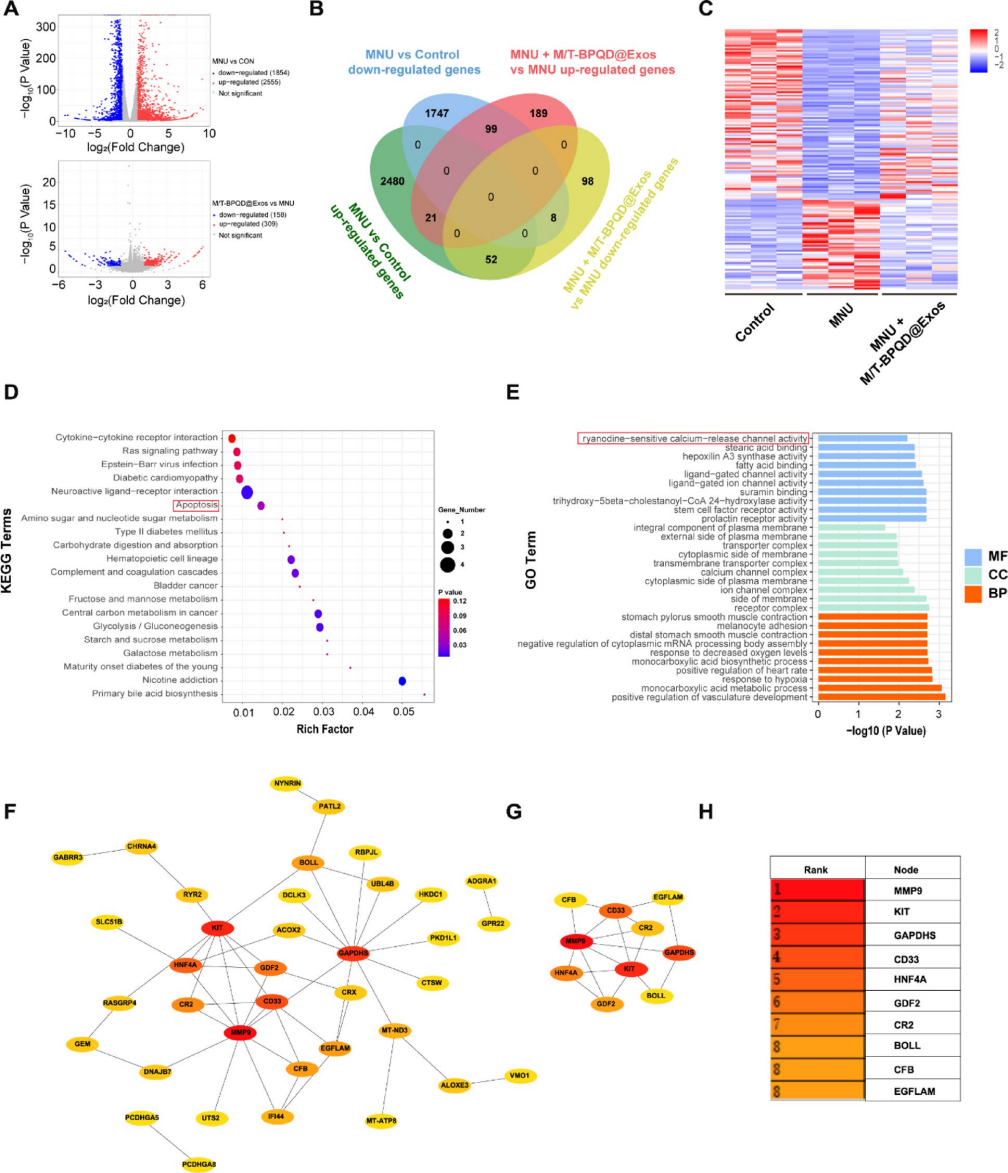
Transcriptomic bioinformatics analysis reveals that M/T‑BPQD@Exos modulate calcium signaling and apoptosis‑related gene networks in photoreceptor degeneration. (**A**) Volcano plots showing DEGs in MNU vs. Control and MNU + M/T-BPQD@Exos vs. MNU comparisons. Red dots represent upregulated genes, blue dots represent downregulated genes, and gray dots represent non-DEGs (|log₂FC| > 1, *P* < 0.05). (**B**) Venn diagram illustrating the overlap of DEGs across MNU vs. Control (up/down) and MNU + M/T-BPQD@Exos vs. MNU (down/up) comparisons. (**C**) Heatmap showing hierarchical clustering of overlap 151 DEGs in Control, MNU, and MNU + M/T-BPQD@Exos groups. Color gradient indicates relative expression levels (red: high, blue: low). (**D**) KEGG pathway enrichment analysis of the 151 DEGs. Bubble size indicates the number of enriched genes, and color gradient indicates the significance (*P* value). (**E**) GO enrichment analysis of the DEGs, categorized by MF, CC, and BP. (**F**) PPI network analysis of the key DEGs. Nodes represent genes, and edges represent functional interactions. (**G**) Module analysis of the PPI network, highlighting the core functional module associated with calcium signaling and apoptosis. (**H**) Ranking of the top 10 hub genes identified from the PPI network, with color gradient indicating gene connectivity (red: high, orange: low). MNU: N-methyl-N-nitrosourea; DEGs, differentially expressed genes; KEGG, Kyoto Encyclopedia of Genes and Genomes; GO, Gene Ontology; PPI, protein–protein interaction; MF, Molecular Function; CC, Cellular Component; BP, Biological Process

Transcriptomic analysis showed MNU induced widespread gene expression changes (2555 up/1854 down), which were partially reversed by M/T-BPQD@Exos (99 up/52 down) (Fig. 5A). Venn diagram analysis identified 151 core genes—comprising 99 genes downregulated by MNU but upregulated by treatment, and 52 genes upregulated by MNU but downregulated by treatment—underscoring the nanocarrier’s key regulatory effects (Fig. 5B). Heatmap analysis demonstrated distinct clustering of gene expression patterns across the control, MNU, and MNU + M/T-BPQD@Exos groups, with MNU-induced expression patterns largely restored by M/T-BPQD@Exos intervention (Fig. 5C). KEGG pathway enrichment analysis revealed that the DEGs were primarily enriched in pathways including cytokine-cytokine receptor interaction, the Ras signaling pathway, and diabetic cardiomyopathy, with apoptosis identified as a key enriched term (Fig. 5D**)**. GO enrichment analysis demonstrated that the DEGs were predominantly associated with molecular function terms related to ryanodine-sensitive calcium-release channel activity and heparin A3 synthase activity **(**Fig. 5E**)**. To further identify the core hub genes, protein-protein interaction network was constructed **(**Fig. 5F**)**, revealing a dense interaction network centered on key genes such as RYR2, MMP9, GAPDHS, and CD33. Module analysis **(**Fig. 5G**)** and hub gene ranking **(**Fig. 5H**)** further screened out top candidate hubs, including KIT, GAPDHS, CD33, and HNF4A, which were verified as the key regulators mediating the protective effects of M/T-BPQD@Exos, primarily through the regulation of calcium homeostasis and apoptotic signaling pathways.

### M/T‑BPQD@Exos inhibits calcium overload and reverses mitochondrial apoptosis in MNU‑damaged photoreceptors

**Fig. 6:**
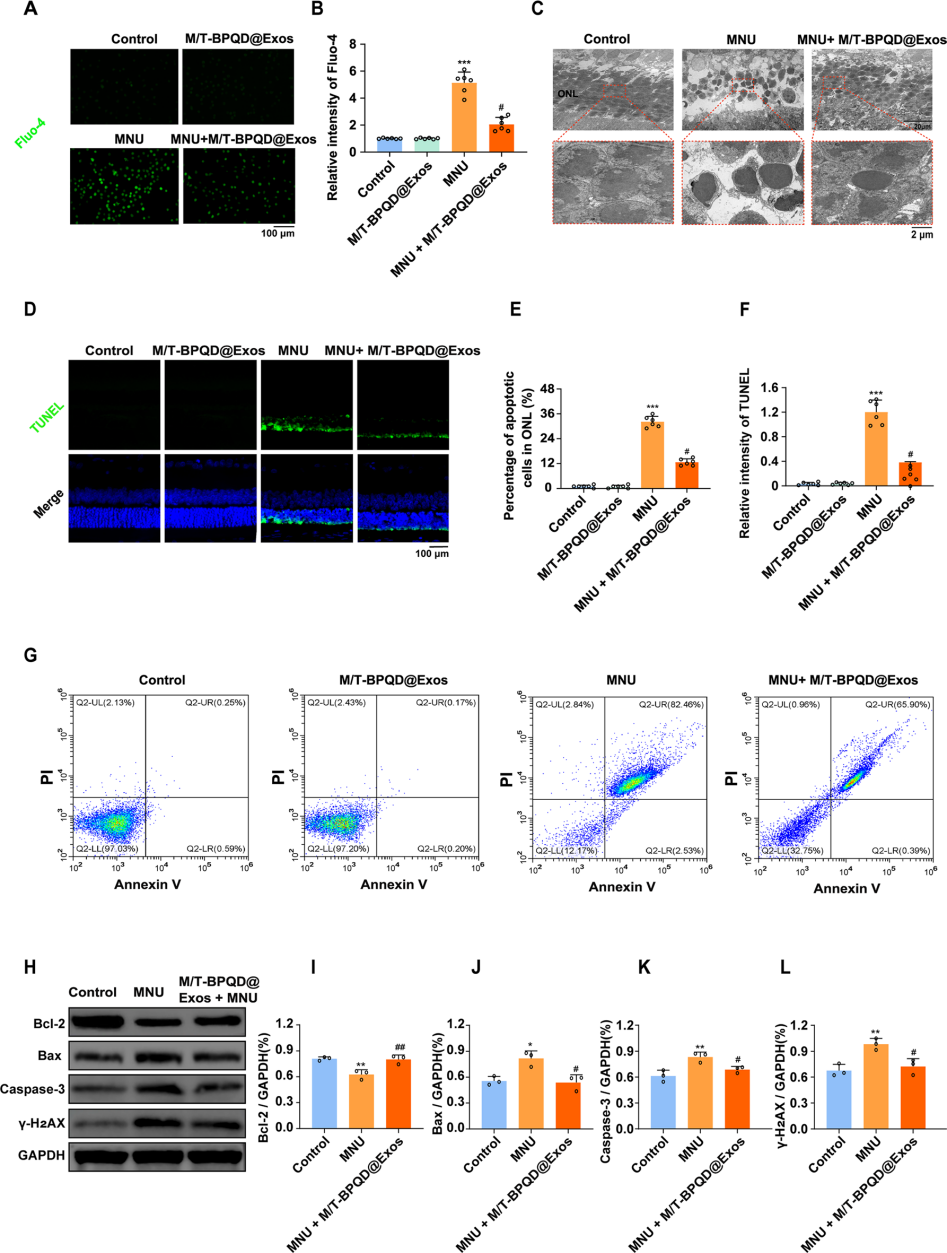
M/T-BPQD@Exos alleviates MNU-induced calcium overload, mitochondrial damage, and apoptotic cell death in photoreceptors. (**A**) Representative Fluo-4 AM fluorescence images showing intracellular calcium levels in 661W cells treated with control, M/T-BPQD@Exos, MNU, and MNU + M/T-BPQD@Exos. (**B**) Quantitative analysis of relative Fluo-4 fluorescence intensity. (**C**) TEM images of retinal photoreceptor ultrastructure in each group, with magnified views of mitochondria. (**D**) Representative TUNEL staining images of rat retinal sections in each group. (**E**-**F**) Quantitative analysis of the percentage of TUNEL-positive apoptotic cells in the ONL and relative TUNEL fluorescence intensity. (**G**) Flow cytometric analysis of apoptotic 661W cells via Annexin V/PI staining, with quadrants representing live cells (Q2-LL), early apoptotic cells (Q2-LR), late apoptotic cells (Q2-UR), and necrotic cells (Q2-UL). (**H**) Western blot analysis of apoptosis-related proteins (Bcl-2, Bax, Caspase-3, γ-H2AX) in 661W cells. (**I**-**L**) Quantitative analysis of Bcl-2, Bax, Caspase-3, and γ-H2AX protein expression levels, normalized to GAPDH. ONL: outer nuclear layer; TEM, Transmission electron microscopy; TUNEL, terminal deoxynucleotidyl transferase dUTP nick end labeling; FITC, fluorescein isothiocyanate; GAPDH, glyceraldehyde-3-phosphate dehydrogenase. Scale bars: 100 μm (A); 20 μm (upper), 2 μm (lower, magnified) (C); 100 μm (D). *p < 0.05, **p < 0.01, ***p < 0.001 vs. Control; ^#^*p* < 0.05, ^##^*p* < 0.01, vs. MNU group

To further delineate the underlying mechanisms by which M/T‑BPQD@Exos protects against MNU‑induced photoreceptor degeneration, we evaluated intracellular calcium homeostasis and apoptotic signaling. Fluo-4 AM staining revealed that MNU treatment induced obvious calcium overload, as evidenced by robust green fluorescence, whereas M/T-BPQD@Exos co-treatment significantly diminished intracellular calcium levels, with quantitative analysis confirming a marked reduction in Fluo‑4 intensity relative to the MNU group **(**Fig. 6A-B). TEM observed typical mitochondrial damage and nuclear pyknosis in MNU group, while mitochondrial structure was well preserved in the treatment group (Fig. 6C). Consistent with calcium homeostasis regulation, TUNEL staining **(**Fig. 6D**)** showed extensive DNA damage in MNU-treated retinas, which was dramatically ameliorated by M/T-BPQD@Exos; quantitative analysis confirmed a significant decrease in the percentage of TUNEL-positive cells in the ONL **(**Fig. 6E) and lower TUNEL intensity (Fig. 6F) compared to the MNU group. Flow cytometric analysis of Annexin V/PI staining further demonstrated that MNU exposure promoted a high rate of early and late apoptosis, whereas M/T-BPQD@Exos treatment shifted the cell population toward live/necrotic profiles, significantly reducing the apoptotic rate relative to MNU **(**Fig. 6G**)**. Western blot analysis revealed that MNU treatment downregulated the anti-apoptotic protein Bcl-2 and upregulated pro-apoptotic markers Bax, caspase-3, and γ-H2AX; in contrast, M/T-BPQD@Exos significantly upregulated Bcl-2 expression and downregulated Bax, caspase-3, and γ-H2AX levels, verifying its robust anti-apoptotic and DNA damage-protective effects **(**Fig. 6H–L**)**. Collectively, these results demonstrate that M/T-BPQD@Exos exerts potent neuroprotection by suppressing MNU-induced calcium overload and preserving mitochondrial integrity, thereby inhibiting mitochondrial-dependent apoptotic cell death.

## Discussion

In this study, we successfully developed a dual-targeted, multifunctional M/T‑BPQD@Exos for the treatment of photoreceptor degeneration. This platform integrates TAT‑mediated retinal penetration, MH42‑mediated photoreceptor targeting, and BPQD‑mediated anti-apoptotic effects into a single biocompatible system. Our results demonstrate that M/T‑BPQD@Exos efficiently penetrates retinal layers, specifically accumulates in the ONL, preserves photoreceptor structure and function, alleviates visual function impairment, and acts by suppressing calcium overload, maintaining mitochondrial integrity, and inhibiting mitochondrial‑dependent apoptosis. This is the first study to combine TAT/MH42 dual targeting with BPQD‑based neuroprotection in engineered exosomes for retinal degenerative diseases, highlighting its novelty and translational potential.

A key innovation of this study is the rational combination of TAT and MH42 peptides to achieve hierarchical targeting. TAT equips exosomes with robust cell-penetrating capacity, enabling efficient traversal of ocular barriers and deep penetration into the retina via the corneal–scleral pathway [26]. Compared with single TAT‑modified carriers, which often lack cell‑specificity and distribute widely across retinal layers, MH42 provides exquisite specificity for photoreceptors, ensuring that the therapeutic payload is concentrated precisely at the site of degeneration rather than being sequestered by RPE cells, Müller glia, or other non‑target cells [17, 27]. Conversely, MH42‑conjugated systems without a penetration peptide often fail to efficiently reach the outer retina due to the resistance of ocular diffusion barriers [16]. Our in vivo retinal distribution results confirmed that unmodified exosomes failed to reach the retina, whereas M/T‑BPQD@Exos efficiently penetrated to the ONL. This dual‑peptide strategy overcomes the two most critical limitations of conventional ocular delivery systems and represents a generalizable platform for retinal targeted therapy.

BPQDs serve as the multifunctional therapeutic core of this system [28]. BPQDs effectively scavenge reactive oxygen species (ROS), alleviate oxidative stress, promote diabetic bone defect healing, and accelerate infected wound repair [19, 29, 30]. Recent studies indicate that the application of BPQDs in ocular diseases is mainly limited to anterior segment disorders. For instance, BPQDs with potent antioxidant activity have been loaded into chitosan nanoparticles (BP@CS) and administered via subconjunctival injection to treat excessive ROS-driven corneal neovascularization after alkaline burn. BP@CS continuously eliminated ROS and markedly suppressed corneal neovascularization, revealing the promising potential of BPQDs in corneal protection [30]. Notably, the use of BPQDs for anti‑apoptotic protection and mitochondrial calcium overload relief in photoreceptors during retinal degenerative diseases has not been reported to date. Collectively, these findings highlight the unique value of BPQDs in mitigating oxidative stress and pathological angiogenesis, yet their potential in preserving photoreceptor function and maintaining mitochondrial homeostasis under degenerative stress remains largely unexplored to date. To fully unlock the therapeutic potential of BPQDs in the posterior segment while overcoming their intrinsic limitations, we rationally integrated them with hDPSC-derived exosomes as a biocompatible and targeted delivery platform. hDPSC-derived exosomes provide intrinsic biocompatibility, low immunogenicity, and natural tissue tropism. Notably, hDPSCs represent an optimal source of exosomes for retinal neuroprotection compared with bone marrow, adipose, or umbilical cord‑derived MSCs [31, 32]. hDPSCs originate from the neural crest and exhibit superior neuroprotective potency: hDPSC-Exos have been shown to protect retinal ganglion cells in glaucoma [33], inhibit neuronal apoptosis in cerebral ischemia/reperfusion injury [34], and promote neurite outgrowth in peripheral nerve injury, which supports their unique advantages in rescuing photoreceptors from degeneration [33]. Moreover, hDPSCs are easily obtained from extracted teeth with non-invasive collection, abundant sources, and no ethical concerns, making them highly translational [23, 35]. The low immunogenicity was further validated by ELISA analysis of serum immune-related cytokines. This characteristic effectively prevents immune rejection and sustained intraocular inflammation after repeated topical administration, which is critical for long-term retinal intervention [36]. Compared with cell therapy and viral vectors that carry high immunogenic risks, hDPSC-Exos exhibit superior clinical safety, laying a solid foundation for future clinical translation [5, 37]. Exosomes were employed as an ideal nanocarrier for BPQD delivery for several critical reasons. First, bare BPQDs may exert cytotoxicity toward vascular endothelial cells and retinal cells which largely limits their clinical application. Exosome encapsulation could effectively reduce the inherent toxicity of BPQDs and improve biocompatibility. Second, BPQDs are prone to oxidation and degradation in physiological environments, while exosome membranes could protect BPQDs from oxidation and enhance their stability. Third, exosomes possess natural membrane fusion properties and superior cell internalization ability, which facilitates the cellular uptake and intracellular delivery of BPQDs. Compared with synthetic lipid or polymeric nanoparticles, which may induce local inflammation or rapid clearance, exosomes enable safer and more sustained retinal retention. By encapsulating BPQDs within exosomes, we improved their stability, prolonged their retinal retention up to 7 days, and protected them from rapid clearance. Our biosafety results confirmed no systemic organ toxicity, no abnormal liver or renal function, and no significant inflammatory responses, supporting the clinical safety of this platform. The biodegradability of BPQDs into non-toxic phosphate ions further eliminates long-term biosafety concerns, a major advantage over many non-degradable inorganic nanomaterials.

Transcriptomic analysis provided unbiased insights into the molecular mechanisms. MNU caused extensive transcriptomic disturbances involving apoptosis, inflammation, and calcium signaling. M/T‑BPQD@Exos treatment reversed these pathological expression patterns and modulated hub genes including MMP9, GAPDHS, CD63, KIT, HNF4A, and CRX. Among these hub genes, MMP9, KIT, and CRX synergistically regulate calcium homeostasis, mitochondrial integrity, and photoreceptor survival [38]. MMP9 is a critical mediator of extracellular matrix degradation, membrane damage, and intracellular calcium overload. Excessive MMP9 disrupts membrane stability, triggers abnormal calcium influx, induces mitochondrial permeability transition, and promotes mitochondria-dependent apoptosis in retinal diseases [39, 40]. KIT encodes a tyrosine kinase receptor that supports cell survival, suppresses apoptotic signaling, and maintains mitochondrial function under stress [41]. CRX is a photoreceptor-specific transcription factor essential for opsin expression, mitochondrial homeostasis, and calcium buffering, whose downregulation exacerbates calcium dysregulation and apoptotic death [42, 43]. In our study, RNA-seq analysis revealed that M/T‑BPQD@Exos significantly downregulated the pro‑apoptotic gene MMP9, while upregulating the pro‑survival gene KIT and the photoreceptor‑maintaining transcription factor gene CRX. These gene expression changes collectively suggest a potential role in suppressing calcium overload, preserving mitochondrial structure, and inhibiting mitochondrial apoptosis. Consistent with our phenotypic observations, these transcriptomic alterations imply that the coordinated regulation of MMP9/KIT/CRX may serve as a key molecular signature underlying the photoreceptor‑protective effects of M/T‑BPQD@Exos.

Despite these promising findings, several limitations should be addressed in future studies. First, the therapeutic window and long‑term efficacy beyond 7 days require further evaluation. Second, the study was performed in a chemically induced MNU model; validation in genetic models of RP (e.g., RCS rats, Pde6b mutant mice) is necessary to confirm generalizability. Third, future studies will optimize dosage regimens, explore non‑invasive eye‑drop administration, and evaluate efficacy in non‑human primates. Finally, the precise receptor targeted by MH42 and the detailed intracellular trafficking pathway of M/T-BPQD@Exos warrant further investigation. Formal pharmacokinetic investigations were not included in this proof-of-concept study, which is an acknowledged limitation, and related profiling will be implemented in our future work.

Despite the above limitations, this photoreceptor-targeted exosome eye drop still exhibits great clinical translational potential. Compared with intravitreal injection, topical instillation is minimally invasive. It possesses favorable retinal targeting ability, long-term ocular retention and excellent biosafety, showing broad application prospects for the treatment of retinal degenerative diseases [44, 45]. Nevertheless, several key challenges still need to be addressed before clinical transformation: (1)large-scale standardized production of engineered exosomes; (2) further optimization of formulation stability suitable for clinical transportation and long-term storage; (3) long-term safety evaluation in non-human primates and other large animal models; (4) standardization of clinical administration dosage and frequency regimens [46, 47].

## Conclusion

We successfully developed a dual-targeted engineered exosome (M/T-BPQD@Exos) co-modified with TAT and MH42 for targeted delivery of BPQDs to treat photoreceptor degeneration. M/T-BPQD@Exos efficiently penetrates retinal barriers, specifically targets photoreceptors, and exerts robust neuroprotection by inhibiting calcium overload, preserving mitochondrial integrity, and blocking mitochondrial-dependent apoptosis. This system significantly preserves retinal structure, restores visual function, improves cognitive outcomes, and shows excellent biosafety. M/T-BPQD@Exos represents a promising and translatable nanotherapeutic strategy for retinal degenerative diseases.

## Supplementary Information

Below is the link to the electronic supplementary material.

Supplementary Material 1.

## Data Availability

No datasets were generated or analysed during the current study.
