# Supplementary material for: Photoreceptor-targeted engineered exosomes encapsulating black phosphorus quantum dots for alleviating photoreceptor degeneration
Source: J Nanobiotechnology. 2026 Jul 8;24:739. doi: 10.1186/s12951-026-04763-x (PMC13459398; doi:10.1186/s12951-026-04763-x)
Supplement: Supplementary file 1 [file 12951_2026_4763_MOESM1_ESM.docx]

Supplementary data

**Title: Photoreceptor-targeted engineered exosomes encapsulating black phosphorus quantum dots for alleviating photoreceptor degeneration**

Authors: Jia Liang^1#^, Xiangqing Hei^2,1#^, Lu Chen^1#^, Zhenhua Zou^1^, Yuke Ji^1^, Lujia Feng^1^, Wangting Li^1^, Yijing Zhuang^1^, Bingyu Bai^1^, Renxue Liang^1^, Yunyun Zou^1^, Pan Xiao^1^, Qiang Lin^1^, Huiyan Zheng^1^, Dong Fang^2,1^*, Wei Chi^1^* and Shaochong Zhang^1^*

^1^ Shenzhen Eye Hospital, Shenzhen Eye Medical Center, Southern Medical University, Shenzhen, Guangdong, China.

^2^ Shenzhen Eye Hospital, Jinan University, Shenzhen, Guangdong, China.

*** Correspondence:**

Shaochong Zhang: Shenzhen Eye Hospital, Shenzhen Eye Medical Center, Southern Medical University, 18 Zetian Road, Shenzhen, Guangdong 518040, China; shaochongzhang@outlook.com; Tel: +86 182-0099-1688

Wei Chi：Shenzhen Eye Hospital, Shenzhen Eye Medical Center, Southern Medical University, 18 Zetian Road, Shenzhen, Guangdong 518040, China; E-mail: chiwei@mail.sysu.edu.cn

Dong Fang：Shenzhen Eye Hospital, Jinan University,18 Zetian Road, Shenzhen, Guangdong 518040, China; E-mail: dora.eye@hotmail.com.

^#^These authors contributed equally to this work

**Supplementary Table 1. Stability evaluation of M/T-BPQD@Exos under different conditions**

| **Storage condition** | **Duration** | **Mean particle size**  **(nm)** | **PDI** | **Morphological observation** |
| --- | --- | --- | --- | --- |
| Initial | 0 d | 110.23 ± 5.17 | 0.15 | Uniform dispersion |
| 4 °C (Short-term) | 7 d | 113.15 ± 6.09 | 0.16 | No aggregation |
| −80 °C (Long-term) | 3 m | 115.43 ± 7.11 | 0.17 | No degradation or aggregation |
| 37 °C (Accelerated) | 24 h | 111.26 ± 5.09 | 0.15 | Good dispersion |

**Fig.S1.** CCK-8-based cell viability detection (OD450 nm). (A) Dose-dependent cytotoxicity of MNU (5 h treatment); (B) Gradient BPQD intervention on MNU-damaged cells; (C) Dose-effect of hDPSC-Exos for MNU injury rescue; (D) Comparison of cytoprotective capacity among hDPSC-Exos, free BPQDs and M/T-BPQD@Exos. **p* < 0.001 vs Control; ^#^ *p* < 0.05, ^##^*p* < 0.01 vs MNU group.

**Fig.S2.** The physicochemical parameters of hDPSC-Exos, BPQD@Exos and M/T-BPQD@Exos (n=3): (A) particle size; (B) zeta potential; (C) PDI. Statistical significance: **p* < 0.05, ***p* < 0.01, ****p* < 0.001.

**Fig.S3.** The cumulative release of BPQDs from M/T-BPQD@Exos in saline solution at pH 7.4.

**Fig.S4.** Comparison of four inflammatory cytokine levels in rat aqueous humor between control and M/T-BPQD@Exos groups. No significant differences of intraocular inflammatory cytokines were observed.

**Fig.S5.** The safety evaluation of M/T-BPQD@Exos in the eye. (A) Fluorescence imaging of ocular surface signals in control and M/T-BPQD@Exos groups at different time points (0 h–7 d); (B) Slit-lamp photographs showing no obvious ocular inflammation or damage in either group.
